# Polythermal structure of a Himalayan debris-covered glacier revealed by borehole thermometry

**DOI:** 10.1038/s41598-018-34327-5

**Published:** 2018-11-14

**Authors:** Katie E. Miles, Bryn Hubbard, Duncan J. Quincey, Evan S. Miles, Tenzing C. Sherpa, Ann V. Rowan, Samuel H. Doyle

**Affiliations:** 1 Centre for Glaciology, Department of Geography and Earth Sciences, Aberystwyth University, Aberystwyth, UK; 2 School of Geography, University of Leeds, Leeds, UK; 3 Department of Environmental Science and Engineering, Kathmandu University, Kathmandu, Nepal; 4 Department of Geography, University of Sheffield, Sheffield, UK

## Abstract

Runoff from high-elevation debris-covered glaciers represents a crucial water supply for millions of people in the Hindu Kush-Himalaya region, where peak water has already passed in places. Knowledge of glacier thermal regime is essential for predicting dynamic and geometric responses to mass balance change and determining subsurface drainage pathways, which ultimately influence proglacial discharge and hence downstream water availability. Yet, deep internal ice temperatures of these glaciers are unknown, making projections of their future response to climate change highly uncertain. Here, we show that the lower part of the ablation area of Khumbu Glacier, a high-elevation debris-covered glacier in Nepal, may contain ~56% temperate ice, with much of the colder shallow ice near to the melting-point temperature (within 0.8 °C). From boreholes drilled in the glacier’s ablation area, we measured a minimum ice temperature of −3.3 °C, and even the coldest ice we measured was 2 °C warmer than the mean annual air temperature. Our results indicate that high-elevation Himalayan glaciers are vulnerable to even minor atmospheric warming.

## Introduction

A glacier’s thermal regime exerts a strong influence on its dynamics, mass balance, and thus its response to climatic change – a particular concern with rising atmospheric temperatures^1,2^. Temperate glacier ice (defined as ice at the melting-point temperature, *T*_*m*_) yields greater ice velocities than cold ice (below *T*_*m*_), both through the more rapid deformation of warmer ice under a given stress, and through basal motion, which is facilitated by the presence of meltwater at the ice-bed interface and within subglacial sediments, if present^3,4^. Temperate ice will also exhibit enhanced ablation rates and yield greater proglacial discharge than cold ice, aided by the increased importance of a subglacial drainage system to evacuate meltwater^5^. Millions of people in the foothills of the Hindu Kush-Himalaya region depend on glacier melt as part of their water resources^6^, yet measurements of the internal characteristics and dynamics of mountain glaciers, particularly their internal temperature field, are scarce. At higher elevations, rising surface temperatures may cause peak meltwater to elapse in the next 30 years, leading to a long-term reduction in the glacial contribution to downstream water resources^1,2,7^. It is therefore increasingly important to determine glacier thermal regimes to better forecast 21^st^ Century glacier retreat and meltwater production.

Most spatially-distributed numerical models of Himalayan glacier motion include only an unrefined representation of glacier dynamics^8^ while, to our knowledge, none includes an empirically-constrained thermal regime^1,9,10^. The only higher-order dynamic model that has been applied to a debris-covered Himalayan glacier^11^ calculated englacial and subglacial temperatures by solving for thermal fluxes^12^ that were estimated in the absence of field data. Consequently, predictions of future mass loss vary and contain large uncertainties; for example, projections of glacier mass loss in the Everest region by 2100 range between 10% and 99%^9–11^.

Debris-covered glaciers have a more complex surface topography and differing mass loss processes compared to clean-ice glaciers^13,14^, complicating direct measurement of internal ice temperature. Seasonal variations in subglacial hydrology inferred from satellite-derived surface velocities suggest the presence of temperate ice at the base of high-elevation debris-covered glaciers^15,16^. Limited field measurements of ice temperatures have been made, but only reached shallow depths (<~20 m) where ice temperature is influenced by seasonal variations in air temperature^17^. Measurements in this zone do not therefore reflect longer-term and deeper ice temperatures. For example, a single temperature measurement of −5.3 °C was made at 2.7 m depth on Khumbu Glacier in 1974^18^; drilling reached 20.3 m depth where the borehole froze shut, which was interpreted to indicate a perennially cold shallow ice zone^19^. A shallow borehole drilled on Rongbuk Glacier (located north of Mt. Everest) in 1966 gave an ice temperature of −4 °C at 3 m depth and −2.1 °C at 10 m depth; this gradient was used to infer temperate ice at depth for other glaciers south and east of Mt. Everest that are at slightly lower elevations^20^. Ice temperatures have been modelled for East Rongbuk Glacier and matched to empirical measurements from three boreholes^21^: one from ice core analysis high in the accumulation area^22^, the other two from shallow boreholes in the ablation area, but no other methodological data were provided^21^. More recent work on four high-elevation Himalayan glaciers found that ice temperature on the Gyabrag Glacier (north-west of Mt. Everest) was −8.0 °C at a depth of 10 m, ~3–4 °C warmer than the mean annual air temperature (MAAT)^23^. However, temperatures remain unknown below the shallow seasonally-influenced layer, particularly at depths where the thermal conditions would be most relevant for modelling ice flow.

**Figure 1:**
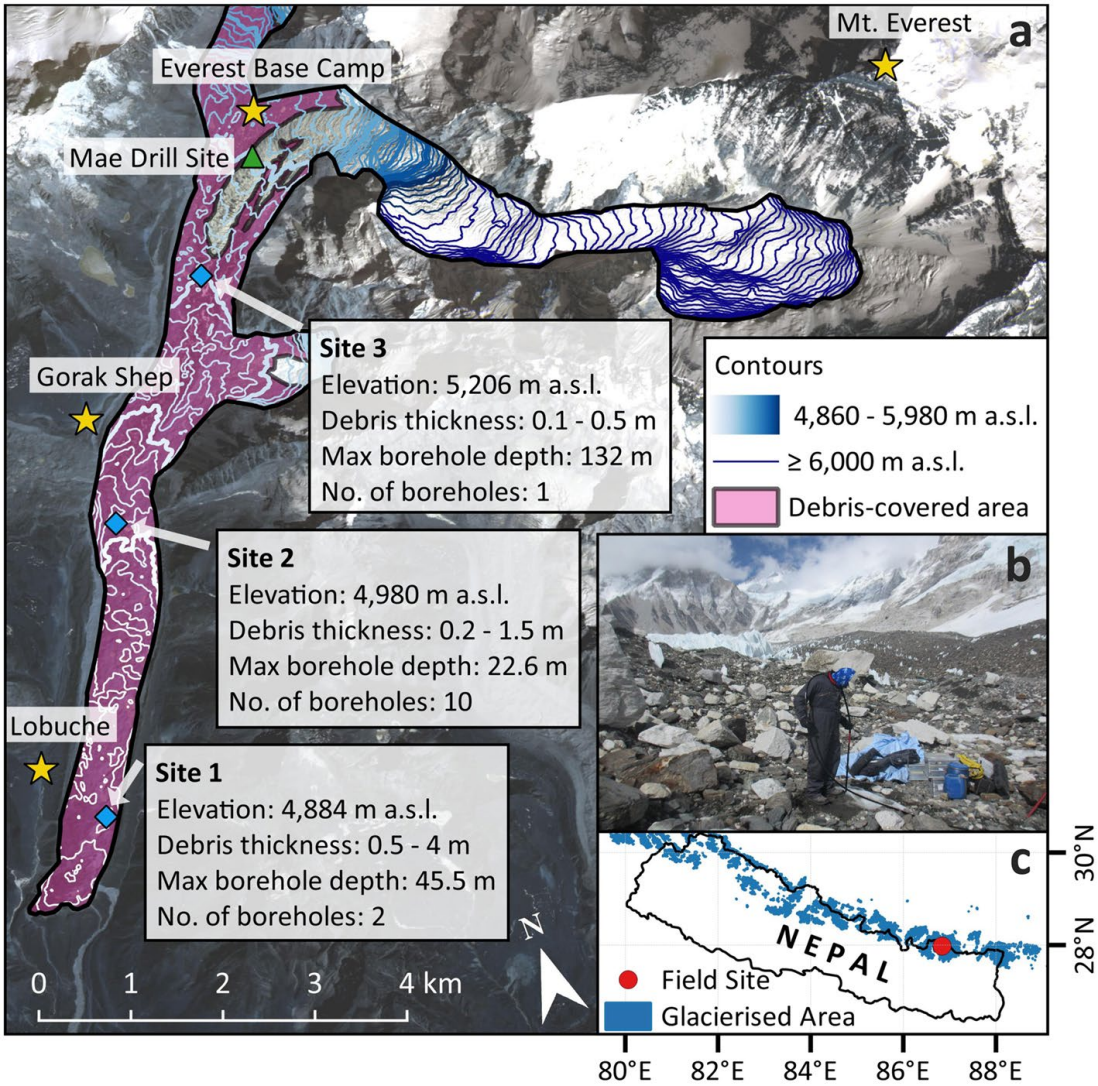
Location map of Khumbu Glacier (**a**) showing the drill sites (blue diamonds) and relevant information about each site from field observations (debris thickness ranges are estimated from field observations). The approximate position of the 1974 Mae drill site^18^ is also marked (green triangle), with villages and Mt. Everest shown for reference (yellow stars). The background image is a RapidEye scene^49^ obtained on 23.02.2017. Contours were created from the 2015 SETSM DEM^50^ and are at 20 m intervals; each 100 m contour within the ablation area is shown in bold from 5,000–5,900 m a.s.l. Inset (**b**) shows an image from Site 3 to demonstrate the glacier surface in the upper part of the ablation area (person drilling shown for scale, image taken by K.M.); inset (**c**) shows the location of the field site (red circle) within Nepal, including the glacierised area across Nepal from the Randolph Glacier Inventory^51^.

Here, we present ice temperature profiles measured along Khumbu Glacier, which originates high on the Nepali side of Mt. Everest and currently terminates at ~4,850 m a.s.l. (Fig. 1). Boreholes were drilled in May 2017 at three locations along the glacier’s ablation area; the deepest at each site was instrumented with a thermistor string (see Methods). At Site 1, the 45.5 m deep borehole was instrumented with nine thermistors; at Site 2, the 22.6 m deep borehole was instrumented with five thermistors; at Site 3, the 132 m deep borehole was instrumented with eleven thermistors. Data were retrieved in October 2017 having recorded englacial ice temperatures during the monsoon and post-monsoon periods^24,25^. Our thermistor naming convention (e.g. S1_5.0) has two parts: ‘S*’ refers to the site; the suffix denotes the depth (in metres) of each thermistor below the surface.

**Figure 2:**
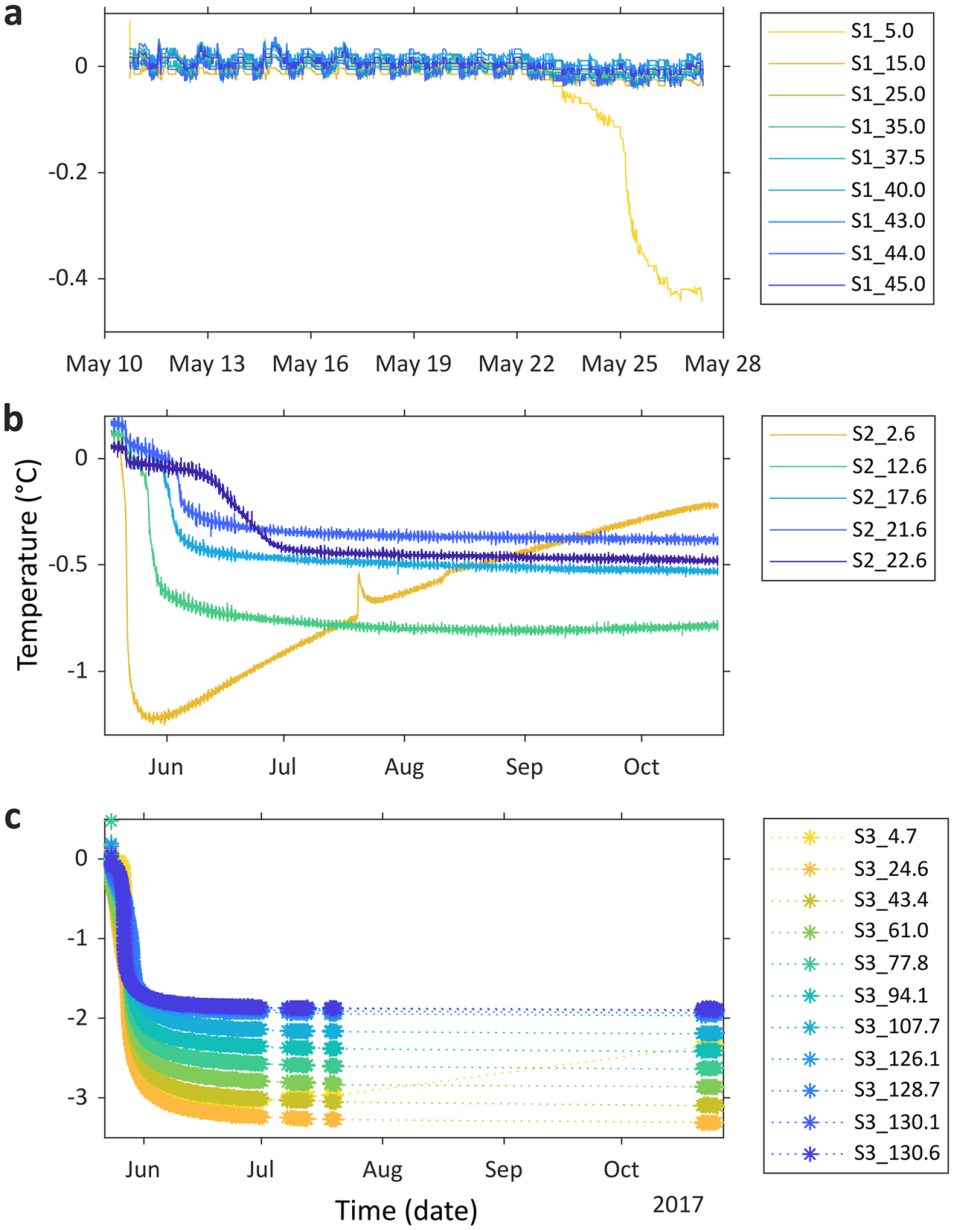
Time series of temperatures measured by each borehole thermistor string for: (**a**) Site 1; (**b**) Site 2; and (**c**) Site 3. Star symbols are used to show data in panel **c** because malfunctioning equipment resulted in some missing data (interpolated using dotted lines); however, all freezing curves were captured. Note the different axis limits on each panel. Thermistors are colour-coded by depth. The small diurnal signal recorded by the thermistors at Site 2 (**b**; <±0.06 °C variation), and to a lesser extent the other two sites (**a**,**c**; <±0.03 °C variation), results from battery voltage noise from the solar regulator operating during daylight hours. Our thermistor naming convention (e.g. S1_5.0) has two parts: ‘S*’ refers to the site at which the borehole was drilled; the suffix denotes the depth (in metres) of each thermistor below the surface.

Borehole temperature time series (Fig. 2) show an initial decrease in temperature measured by all thermistors at Sites 2 and 3, and the uppermost thermistor at Site 1 (S1_5.0), which we interpret as the freezing of each thermistor into the borehole as the heat injected during drilling is dissipated^26^. Beneath this curve, thermistors settle towards the undisturbed temperature of the surrounding ice^26,27^. Ice temperatures range from −0.47 °C (S1_5.0) to −3.3 °C (S3_24.6). Ice is warmer at Site 1 than Site 3, and in general becomes warmer with depth along each borehole. The uppermost thermistors at Sites 2 and 3 (S2_2.6 and S3_4.7) record increasing temperatures between June and mid-October.

**Figure 3:**
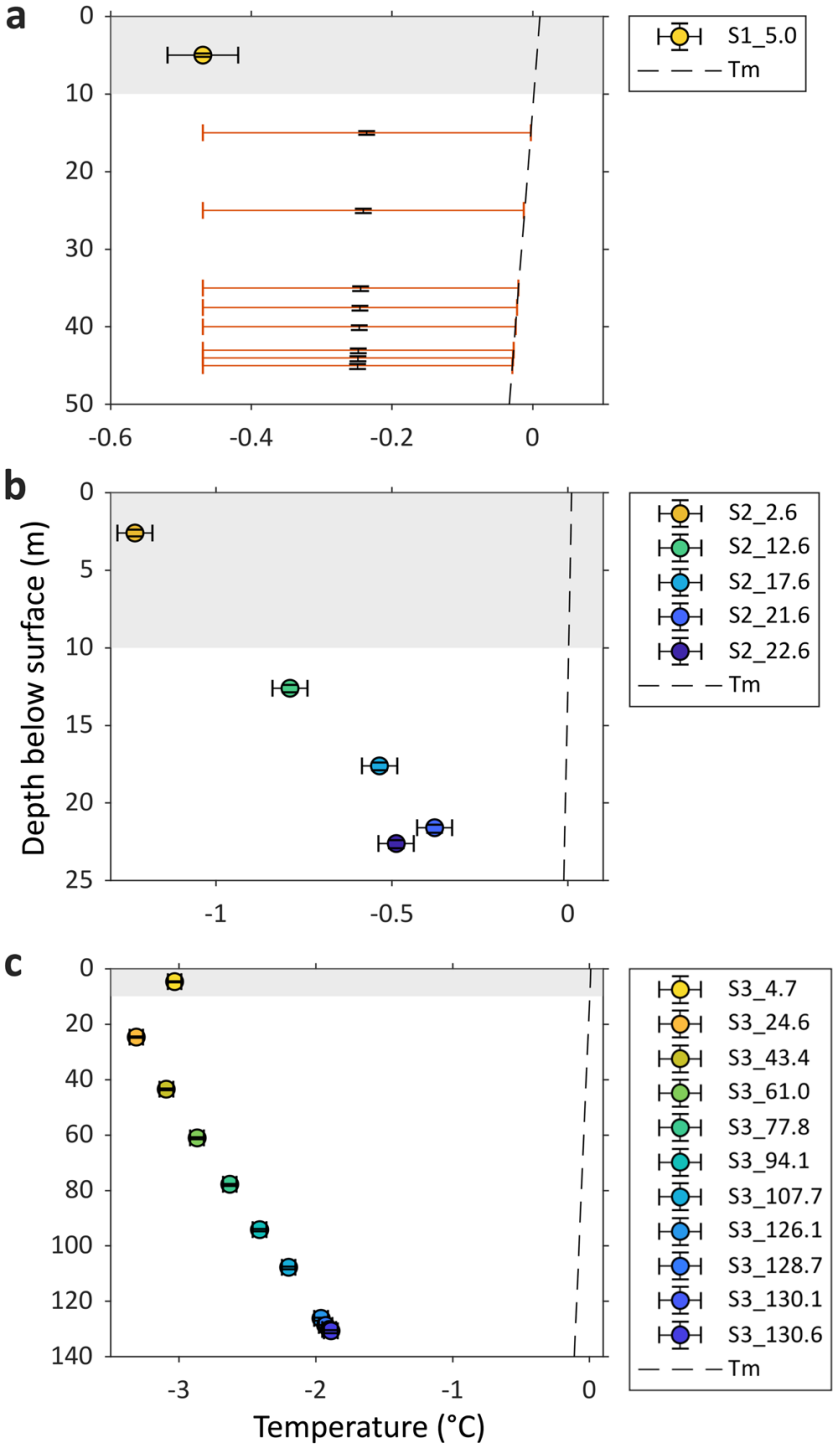
Vertical ice temperature profiles for each borehole thermistor string at: (**a**) Site 1; (**b**) Site 2; and (**c**) Site 3. Note the different axis limits on each panel. Error bars indicate estimated uncertainty in thermistor temperatures (horizontal), which are accurate to ±0.05 °C at 0 °C, and depths (vertical; see Methods). Panel (**a**) shows additional error bars in red to indicate the potential ice temperature range of the thermistors that did not freeze in and only recorded the borehole water temperature (as they did not freeze in before S1_5.0, the ice is inferred not to be colder than the ice around this thermistor; see Text). A dashed line indicates the melting-point temperature (*T*_*m*_; see Methods). Thermistor naming convention is outlined in Fig. 2, and the thermistor colour-coding by depth matches that in Fig. 2. The grey bands mark the 10 m shallow ice layer that is expected to be influenced by seasonal variations in air temperature^17^.

**Figure 4:**
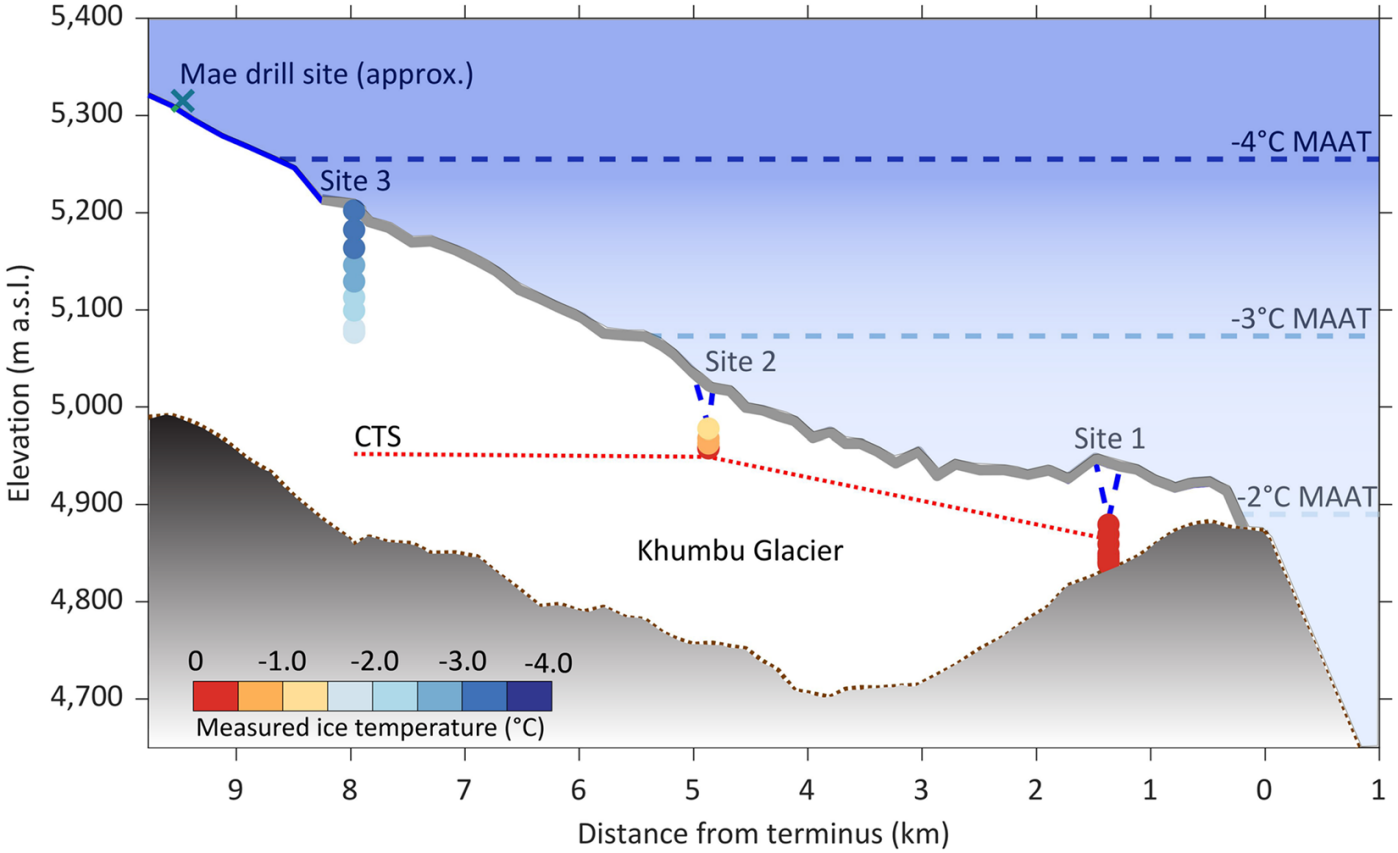
Illustrative long profile of Khumbu Glacier showing the ice temperature values recorded by each thermistor within the three boreholes. The ice surface from the 2015 SETSM DEM^50^ was plotted along the approximate centreline of the glacier (blue line), with the modelled surface debris layer^11^ indicated by a grey line. A blue dashed line has been used to indicate the local borehole elevations in May 2017. It should be noted that the Site 1 borehole was drilled off the centreline, between two supraglacial ponds in a large surface depression. The bed depth was estimated using the SETSM DEM and modelled ice thicknesses^11^ and is plotted with a brown dotted line; the proglacial extent was interpolated between two measured points. It is not known whether the Site 1 borehole reached the bed. The blue dashed MAAT isotherms were calculated from the 1994–2013 lapse rate up the Khumbu Valley^31^. The CTS, estimated from the thermistor data, is plotted as a red dotted line with cold ice above and temperate ice below.

Undisturbed ice temperatures were calculated for each thermistor that showed a freezing curve, and are presented in Fig. 3 with the expected *T*_*m*_ (see Methods). Ice temperatures are generally colder near to the glacier surface and increase approximately linearly with depth (Site 2 *R*^2^ = 0.9206; Site 3 *R*^2^ = 0.9996). All ice temperatures at Site 3 (Fig. 3c) are colder than the coldest measured at Site 2 (Fig. 3b), which are very close in temperature to *T*_*m*_ (a conservative estimate; see Methods). These temperatures are plotted along Khumbu Glacier from the icefall to the terminus (Fig. 4), illustrating the general increase in the temperature field both with depth and towards the terminus. The estimated cold-temperate transition surface (CTS; see Methods)^28^ is included to show the extent of cold and temperate ice within the ablation area.

At Sites 2 and 3, freezing curves initiate within 3 days of thermistor installation due to the rapid dissipation of heat within cold ice (Fig. 2). In contrast, thermistors between 15 and 45.3 m depth at Site 1 show no freezing curves over the 17 days of data available (Fig. 2a) before the cable ruptured due to debris movement, and are interpreted to have recorded only water temperature during this period. We can, however, infer that the ice between thermistors S1_15.0 and S1_45.3 is warmer than that around the uppermost thermistor at Site 1 (S1_5.0); if the deeper ice were as cold, these thermistors would have frozen in over a similar time frame. The potential temperature ranges for ice at the depths of these thermistors is indicated by the red range bars in Fig. 3a. All are warmer than −0.46 °C (S1_5.0), and we thus expect this ice to be very close to, or at, *T*_*m*_^29^.

The ice surrounding the uppermost thermistors at Sites 2 and 3 (S2_2.6, S3_4.7, and S2_12.6 to a much smaller degree) is influenced by seasonal air temperature variations, responding to rising air temperatures through the monsoon (the greater depth of S2_12.6 results in a lag, with warming only beginning in September). Therefore, despite the debris layer, at least the uppermost 10 m of the ablation area is notably influenced by seasonal surface temperatures^17^, similar to clean-ice glaciers^23,30^. The uppermost thermistor at Site 2 (S2_2.6) is most strongly seasonally influenced, reflecting its shallow location and thinner overlying debris layer (Fig. 1), showing a temperature increase of ~1 °C from June to October (Fig. 2b). The sharp rise and more gradual fall in temperature towards the end of July is likely a result of water breaking into the borehole near this thermistor, possibly through a crack, and subsequently either seeping out the borehole or, more likely, cooling and freezing within it.

Our ice temperature measurements reveal that Khumbu Glacier is polythermal; with cold ice in the upper part of the ablation area and temperate ice at depth in the lower part of the ablation area (Fig. 4). The coldest ice temperatures were measured near the surface, but beneath a seasonally influenced upper layer of ~10 m depth. The temperature reversal at the base of the Site 2 borehole (Fig. 3b) may contradict the latter point if the temperature continues to decrease beyond our borehole depth. Indeed, similar minor reversals have been reported elsewhere, but with no explanation^27^. If this overturning is real, the mechanism remains to be explained, but is most likely to be related to the advection of a relatively cold ice layer.

Assuming that the ice does remain temperate to the bed, we estimate that the CTS is at 20 m depth (or shallower) at Site 1, 31 m depth at Site 2, and 255 m at Site 3. This suggests a substantial layer of temperate ice, similar to results from lower-elevation valley glaciers elsewhere in the world^17,27,29^. The temperate layer on Khumbu Glacier would thus comprise ~56% of the ablation area ice volume (Site 3 to terminus). The accumulation area of Khumbu Glacier is above 6,200 m a.s.l. in the Western Cwm of Mt. Everest where the MAAT is below −9 °C^31^. Thus, ice is expected to form at or below −9 °C, as on the northeast side of Mt. Everest^22^. As this cold ice is advected downglacier, it will be warmed from below by geothermal heating, from above by warm air at lower elevations (the Khumbu ablation area is between ~5,300 and 4,850 m a.s.l.), and from within by deformation, refreezing and meltwater flow^17,32,33^. The emergence of ice in the lower part of the ablation area removes colder near-surface ice^17^. These processes explain the generally increasing ice temperatures downglacier and at depth (Fig. 3).

Our near-surface thermistor located at Site 3 (S3_4.7) may be compared with the single nearby measurement of −5.3 °C recorded at 2.7 m depth and at a similar time of year to our measurements (late November, with borehole drilled in August) in 1974^18^. As this measurement was taken in the shallow ice layer affected by seasonal temperature variations, a temperature recorded in November (shortly following the warm season^24^) likely represents close to the warmest temperature the ice reaches during the year. Our equivalent thermistor in this layer (S3_4.7) recorded a minimum temperature of −3.1 °C in late June and a maximum of −2.3 °C in late October at the end of the warm season (Fig. 2c). Assuming similar seasonal surface heat transfer to ~5 m depth in both 1974 and 2017, comparison of these values could indicate a warming of the ice by ~2–3 °C in this area of the glacier in the 43 years between the observations. However, the difference could also be a result of differences in ice advection pathways between the sites, or measurement uncertainty (which is not presented for the Mae data).

Immediately below the uppermost layer, where ice is no longer influenced by seasonal surface temperatures, the undisturbed ice temperatures are warmer than the MAAT by as much as 2 °C (Fig. 4) suggesting additional warming of englacial ice beyond atmospheric heating inputs. Little is known about the thermodynamics of ice transport through icefalls, but if the primary stratification is deformed or latent energy released as meltwater penetrates crevasses and refreezes^34^, this might partially explain the presence of warmer ice at depth farther downglacier. Alternatively, amplified climate warming at high elevations^1,2^ may be penetrating deeper into the glacier. Since ice located above the CTS at Site 2 was within 0.8 °C of *T*_*m*_, and at Site 1 all measurements were within 0.5 °C of *T*_*m*_ (Fig. 3), it is highly likely that towards the terminus the ice temperature is near, if not already at, *T*_*m*_. This is despite the supraglacial debris layer acting to insulate shallow ice temperatures from atmospheric warming. However, the presence of supraglacial ponds^35,36^ hosting bare-ice cliffs, which are subject to thermal erosion^37^, appears to at least counteract the insulating effect of the surface debris layer at Khumbu Glacier.

Our measurements of the thermal regime of Khumbu Glacier have important implications for the future of Himalayan glaciers. Temperate shallow ice located near the terminus, where there are already increasingly large areas of supraglacial ponds^36^, could contribute to more rapid pond expansion^29,38^, increased ice mass loss and water storage within the supraglacial hydrological system^39,40^. If high melt rates continue, the CTS may become shallower as the volume of temperate ice expands, resulting in more energy absorption contributing directly to melt rather than warming cold ice. A layer of warm ice at depth, particularly if it extends to the bed, would allow a widespread englacial and/or subglacial drainage system to persist, potentially enhancing glacier velocity, ablation and water storage, all of which would influence downstream water delivery.

Our analysis of borehole-based ice temperatures within Khumbu Glacier indicates a polythermal regime with ~56% of the ice column being temperate in the lower 8 km of the ablation area. Even in the upper part of the ablation area, ice temperatures are no more than 3.5 °C colder than *T*_*m*_, are up to 2 °C warmer than the MAAT and may be ~2 to 3 °C warmer than ~40 years ago. These data are the first of their kind for this region and for any debris-covered glacier, and will improve predictions of glacier response to climate change and their contribution to downstream water resources. The prevalence of temperate and warming ice at high elevations, even beneath thick supraglacial debris, indicates that these glaciers are highly vulnerable to 21^st^ Century climate warming.

## Methods

### Data collection - boreholes

Thirteen boreholes were drilled into Khumbu Glacier in May 2017 at three sites in the ablation area (Fig. 1) using a pressurised hot-water drilling system^41^ adapted for operation at high elevations. Sites were selected based on proximity to a water supply (a supraglacial pond) and a thin (<0.5 m) local debris layer that could be cleared prior to drilling. At Sites 1 (2 boreholes) and 2 (ten boreholes), drilling ceased due to the presence of debris in the borehole. At Site 1, we believe that the drill may have reached the bed, but this is difficult to confirm with the available observations. At Site 2, ten boreholes were drilled to 12–22 m depth at locations with surface elevation varying by ~10 m, suggesting a spatially extensive and possibly continuous debris layer beneath the surface. The borehole at Site 3 was drilled to the maximum length achievable (~155 m) with our equipment at 5,200 m elevation. Borehole inclinometry revealed that it was drilled off-vertical (reading a maximum of 30° at the base). Thus, although the borehole length was 155 m, the depth of the borehole base was 132 m beneath the glacier surface. The sensor depths presented here have been corrected to reflect the true depth (rather than borehole length). Inclinometer data were not available for the boreholes at Sites 1 and 2, but the deviation at Site 3 is lower towards the surface so no thermistor at Sites 1 or 2 is likely to be more than ~1 m in error.

### Data collection - thermistors

The longest borehole at each site was selected to be instrumented with strings of thermistor sensors. The thermistor string contained negative temperature coefficient thermistors (Honeywell UNI-curve 192-502-LET-AOI) connected by a multicore cable, spaced more closely at depth. Higher up the cable, thermistors were spaced more evenly (typically 10–20 m apart) according to the expected length of the borehole which was based on measured^42^ and modelled^11^ ice thicknesses. Thermistor resistance was measured every 10 minutes with Campbell Scientific CR1000 data loggers, using a half-bridge relative to a precision reference resistor with a low temperature coefficient (15 ppm/°C). Resistance was converted to temperature using a Steinhart and Hart^43^ polynomial fitted to the manufacturer’s calibration curve, with a further correction using a freezing-point offset for each thermistor obtained from an ice-bath calibration. Previous studies using such thermistors^44–46^ suggest that with this secondary calibration, an accuracy of ±0.05 °C can be achieved. We therefore consider the thermistors to be accurate to ±0.05 °C at 0 °C, but accept that this value represents an indication of uncertainty rather than a maximum limit.

Uncertainty in vertical depth for each thermistor was estimated as the sum of error in the exact location of cable splicing (±0.2 m) and cable stretch upon lowering into each borehole (+0.5% of sensor depth). These are indicated in Fig. 3 as vertical error bars. The average depth uncertainty range was 0.66 m, with a maximum depth uncertainty range of 1.05 m (sensor S3_130.6).

### Undisturbed ice temperatures

The undisturbed ice temperature for each thermistor that froze in was estimated by taking the minimum of the running mean for one hour during the night (to avoid the slight noise-related influence from solar charging during the day), late in the time series (to ensure the settled temperature was as close to the true temperature as possible). Some thermistors, for example at Site 2 (Fig. 2b), still show a very slight cooling trend, but this is of the order of hundredths of a degree and is therefore not expected to significantly change the undisturbed ice temperatures we calculate. For the uppermost thermistors at these sites that were influenced by rising surface temperatures during the monsoon (S2_2.6 and S3_4.7), an hour during the night at the lowest point of the freezing curve was used, before the temperature began to rise. At Site 1, the CR1000 was detached from the thermistor string and removed from the site at the end of the May field season due to developing slope instability near the borehole, relating to pond expansion. During the return trip in October, an attempt was made to reconnect the strings to the CR1000, but the cable had been severed by debris and no further data were collected from Site 1. Only the surface-most thermistor at Site 1 (S1_5.0) froze into the borehole, but the full settling curve was not captured before the CR1000 was removed. The undisturbed ice temperature of this thermistor was calculated by using the following equation^26,27^ fitted to the raw thermistor data shown in Fig. 2a:

$$T(t)=(\frac{Q}{4\pi k(t-s)})+{T}_{0}$$

where *T* is the borehole ice temperature at time *t*, *Q* is the heat released by drilling per unit length of the borehole; *k* is the thermal conductivity of pure ice at 0 °C (2.1 W m^−1^ K^−1^), *T*_0_ is the undisturbed ice temperature, and *s* is the time in seconds until the start of the freezing curve.

### Melting-point temperatures

The pressure-dependent melting-point temperature, *T*_*m*_, was calculated at the depth of each thermistor using the Clausius-Clapeyron equation of melting-point depression^27,46^:

$${T}_{m}={T}_{tp}-\gamma \,(\rho -{\rho }_{tr})$$

where *T*_*tp*_ and *ρ*_*tr*_ are the triple point temperature (273.16 K) and the pressure of water (611.73 Pa) respectively, *γ* is the Clausius-Clapeyron constant and *ρ* is the ice overburden pressure, which can be approximated as:

$$\rho ={\rho }_{i}\,g\,h$$

where *ρ*_*i*_ is the density of ice (900 kg m^−3^), *g* is the gravitational acceleration (9.81 m s^−2^) and *h* is the height of the overlying ice column given here by sensor depth (m). Values of the Clausius-Clapeyron constant range from that of pure (air-free) water/ice (0.0742 K MPa^−1^)^30^, a small content of soluble impurities and air within ice (0.079 K MPa^−1^)^47^ to that for pure ice and air-saturated water (0.098 K MPa^−1^)^48^. These values were all tested for the best match of each *T*_*m*_ to the freezing-point of each thermistor^27^: the constant for pure ice and air-saturated water provided the closest fit and was used in the analysis here. However, the theoretical *T*_*m*_ values still differed from the true freezing temperature of the thermistors by 0.04 to 1.1 °C, suggesting that a further factor depressed the freezing-point. One possible explanation is the presence of solutes and impurities within the ice, the concentrations of which are currently unknown within Khumbu Glacier.

### Cold-temperate transition surface

The values of *T*_*m*_ were used along with the undisturbed ice temperatures to estimate the CTS depth^27,28,46^. At Sites 2 and 3, a line of best fit through all the undisturbed thermistor values (as presented in Fig. 3) beneath the surface 10 m was extended until it intersected *T*_*m*_. Goodness-of-fit was calculated to be *R*^2^ = 0.9206 for Site 2, and *R*^2^ = 0.9996 for Site 3. The CTS is interpreted to occur at this intersection of the extended best-fit line with *T*_*m*_, and occurred at a depth of 255 m below the surface at Site 3, and 31 m below the surface at Site 2. To estimate the CTS depth at Site 1, a line of the same gradient as for Site 2 was extrapolated from the mid-point of the error bar for the first thermistor below the seasonally-affected shallow ice layer (S1_15.0). This line intersected *T*_*m*_ at 20 m depth. For reference, when a line of the same gradient as Site 3 was used, it intersected *T*_*m*_ at 30 m depth.

## Data Availability

The datasets presented in this study are available for download from: 10.6084/m9.figshare.7165531.v1.
